# Location Dependence of Mass Sensitivity for Acoustic Wave Devices

**DOI:** 10.3390/s150924585

**Published:** 2015-09-23

**Authors:** Kewei Zhang, Yuesheng Chai, Z.-Y. Cheng

**Affiliations:** 1 School of Materials Science and Engineering, Taiyuan University of Science and Technology, Taiyuan 030024, China; E-Mails: zhangk1@tigermail.auburn.edu (K.Z.); cys5912@126.com (Y.C.); 2 Materials Research and Education Center, Auburn University, Auburn, AL 36849, USA

**Keywords:** mass sensitivity, acoustic wave device, point mass, biosensor, microcantilever, magnetostrictive particles

## Abstract

It is introduced that the mass sensitivity (*S_m_*) of an acoustic wave (AW) device with a concentrated mass can be simply determined using its mode shape function: the *S_m_* is proportional to the square of its mode shape. By using the *S_m_* of an AW device with a uniform mass, which is known for almost all AW devices, the *S_m_* of an AW device with a concentrated mass at different locations can be determined. The method is confirmed by numerical simulation for one type of AW device and the results from two other types of AW devices.

## 1. Introduction

Acoustic wave (AW) devices have been widely studied and used as sensor platforms for the development of various sensors, including physical, biological, and chemical sensors [1,2,3,4,5]. Various AW devices, such as bulk and micro- or nano-AW devices with different geometries, have been developed for these purposes [1,2]. Although the geometries of AW devices can be very different, the principle of all AW devices as sensor platforms is the same [1]: the resonant frequency of an AW device changes with the influence of the environment, such as viscosity, temperature, friction force, and mass load. For the comparison and performance/fundamental study of AW devices, a mass sensitivity (*S_m_* = −Δ*f*/Δ*m*, in the unit of Hz/g) is introduced to characterize the capability of AW devices as sensor platforms. The *S_m_* is defined as the shift in the resonant frequency (Δ*f*) due to the attachment of a mass load (Δ*m*) onto the surface of the AW device [6]. If the mass load is uniformly distributed over the entire surface of an AW device, it is obtained for most of the AW devices that [6,7]:

(1) $$S_{m}^{uni}=-\frac{\Delta f}{\Delta m}\approx \frac{f_{0}}{2M}(\Delta m<<M)$$

where *M* and *f_0_* are the mass and the resonant frequency of the AW device without a mass load. However, the mass load is usually localized (*i.e.*, the mass load is not uniformly distributed) on the surface of an AW device for most applications. Therefore, from an application point view, it is of interest to know the *S_m_* of an AW to a concentrated mass at different locations.

It is well known that the *S_m_* of an AW device is strongly dependent on the location of the mass load [1,8]. That is, for the same mass load attached to the surface of an AW device, the change in the resonant frequency of the AW device is dependent on where (the location) the mass load is. For example, if the mass load is at the node point of an AW device, a zero frequency change (*i.e.*, zero *S_m_*) is expected. In other words, the change in the resonant frequency of an AW device is dependent on both the mass load and the location of this mass load. Therefore, it is critical to know the relationship between the mass sensitivity and the location of the mass load in order for anyone to use the AW device to determine/measure the mass load based on the change in the resonant frequency. Unfortunately, there are no results on how the location will affect the *S_m_* due to the fact that there is no analytic solution.

For some classic applications, the influence of the environment on the surface of an AW device is uniform. For example, the well-known quartz microbalance is widely used in the characterization/measurement of the thickness of a thin film, where the mass load is a uniformly distributed layer over the sensor surface. In this case, the widely-used mass sensitivity is actually defined as the sensitivity with a unit of Hz/(g/cm^2^) for measuring surface density rather than the sensitivity with a unit of Hz/g for measuring the mass itself [1,2]. Even for these widely used AW devices, the mass sensitivity for a concentrated mass is unknown.

In recent years, AW devices have been extensively studied for the development of biological and chemical sensors due to the fact that the AW devices, as a sensor platform, exhibit an unprecedented sensitivity and can be easily miniaturized [9,10]. As demonstrated by the experimental results and indicated by the theoretical research, biosensors using AW devices as sensor platforms show many advantages, especially for the detection of bacteria and viruses, over biosensors using electrochemical and optical devices as sensor platforms [11]. When the biosensors based on AW devices are used for the detection of bacteria, the bacterial cells are bound on the sensor surface as individual particles rather than a uniform thin layer. In this case, the mass load (*i.e.*, bound bacterial cells) is localized. Therefore, the change in the resonant frequency due to a bacterial cell bound on the surface is strongly dependent on the location of the cell. To estimate the bound bacterial cells on the sensor surface by using the measured change in the resonant frequency, it is important to know the relationship between the *S_m_* and the mass-load location. Additionally, if the location dependence of the *S_m_* can be quantified, the AW devices can be developed to measure nanoparticles and the interaction between different nanoparticles. Knowing the location dependence of the *S_m_* would also make it is possible to optimize/enhance the performance of the sensors based on AW devices. For example, since it is known that the *S_m_* reaches its maximum when the mass load is at the free end of a cantilever, a viscosity sensor was developed using a cantilever [12].

Although the location dependence of the *S_m_* is important, it is not known for all AW devices since there is no analytic solution for the location dependence of the *S_m_* for any AW device [1,5]. Here, a new methodology is introduced to simply determine the *S_m_* for a concentrated mass at different locations. The methodology is first proposed based on the numerical simulation of a typical kind of AW device—freestanding magnetostrictive strips, also known as magnetostrictive particles (MSPs) [13,14]. The methodology is then used to determine the location dependence of the *S_m_* for two other types of AW devices—cantilever and diaphragm. The results from this new methodology are compared with, and confirmed by, the results obtained for a special point on a cantilever and diaphragm using the effective mass approach with some approximations: the *S_m_* for a concentrated mass at the free end of a cantilever and the *S_m_* for a concentrated mass at the middle of a diaphragm.

## 2. Theoretical Consideration and Numerical Simulation

MSPs have been developed as a type of AW device for the development of high-performance sensors due to the fact that the MSPs, as a sensor platform, exhibit some unique advantages over other AW devices [11,13]. An MSP is a strip of a magnetostrictive material, whose length changes when subjected to a magnetic field. Sensors using MSPs as sensor platforms exhibit high performance [11,12,13,14,15,16,17]. For example, for the *in situ* and real-time detection of bacteria in water, a detection limit less than 10^2^ cfu/ml has been obtained for the detection of different pathogenic bacteria in liquid [11].

For an MSP with a length of *L*, a width of *W*, and a thickness of *H*, its fundamental resonant frequency (*f*_0_) for the longitudinal vibration is [8,18]:

(2) $$f_{0}=\frac{1}{2L}\sqrt{\frac{E}{\rho \left(1-\sigma \right)}}(L>>W>>H)$$

where, *E*, *ρ* and *σ* are the Young’s modulus, density, and the Poisson’s ratio of the magnetostrictive material, respectively.

If a concentrated mass is attached on to the surface of an MSP, the resonant frequency cannot be obtained with an analytic solution. For numerical simulation, assume an MSP is a plane-stress dominated isotropic elastic plate so that the *E*_plane-stress_ = *E*/(1 − *σ*). When an MSP with a concentrated mass is excited to resonate along the x-axis (*i.e.* length direction), the kinetic energy (*T*) and potential energy (*V*) of the MSP can be expressed as:

(3) $$T=\frac{1}{2}\int_{0}^{L}\rho \cdot A_{s}\cdot {\left(\frac{\partial u\left(x,t\right)}{\partial t}\right)}^{2}dx+\frac{1}{2}\Delta m\cdot {\left(\frac{\partial u\left(x,t\right)}{\partial t}\right)}_{{x=x}_{c}}^{2}$$

(4) $$V=\frac{1}{2}\int_{0}^{L}\frac{E}{1-\sigma }A_{s}{\left(\frac{\partial u\left(x,t\right)}{\partial x}\right)}^{2}dx$$

where, *t* is the time; Δ*m* is the concentrated mass load at a location *x_c_* (0 ≤ *x*_c_ ≤ *L*); *A_s_* is the cross-sectional area (*W × H*) of the MSP; *u*(*x*,*t*) is the displacement vector along the *x*-axis; the first and second terms on the right side in Equation (3) represent the kinetic energy for the MSP and the Δ*m*. The *u*(*x*,*t*) can be expressed as: *u*(*x*,*t*) = φ(*x*)·*q*(*t*), where *u* = [*u*_1_, *u*_2_, *…*, *u_n_*], *u_n_* is the displacement for the *n*th order resonance. *φ*(*x*) = [*φ*_1_, *φ*_2_, …, *φ_n_*] is the mode shape vector, where *φ_n_*(*x*) is the mode shape function of the *n*th order resonance. *q*(*t*), generalized coordinately, is an *n* × *n* matrix. Therefore, the governing vibration equation is derived to determine the *n*th order resonant frequency (*f_n_*) of an MSP with a concentrated mass load. Based on these, and using the same procedure as described by Zhang *et al.* [19], the numerical simulation can be carried out to determine the resonance frequency of an MSP with a concentrated mass load. For the numerical simulation, MATLAB software was used with the properties and dimension of the MSP listed in Table 1.

**Table 1:** Parameters for the MSP used in the numerical simulation.

	Symbol	Unit	Value
Young’s modulus	*E*	GPa	10^5^
Density	ρ*_s_*	kg/m^3^	7.9 × 10^3^
Poisson’s ratio	*ν*	-	0.33
Length	*L*	mm	1
Width	*W*	mm	0.2
Thickness	*H*	μm	15

The properties listed in Table 1 are the same as those of Metglas^TM^ 2826 MB [20], which is widely used in the development of MSP-based sensors. Using Equations (1) and (2) with the properties and dimension listed in Table 1, it is obtained that the fundamental resonant frequency (*f*_0_) is 2.22697 MHz and the $S_{m}^{uni}=46.98\;\text{Hz}/\text{ng}$.

If Δ*m* = 0, the numerical simulation results in a *f*_0_ of 2.22697 MHz, which is the same as what was obtained from Equation (2). This also shows the correctness of the numerical simulation approach used here.

**Figure 1:**
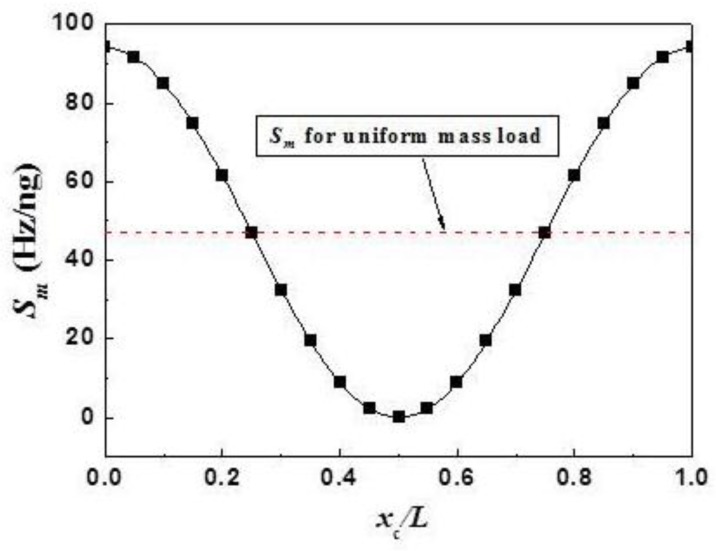
Simulated *S_m_* (solid squares) for the concentrated mass load at different locationsm (*x_c_*) for the MSP operated at the fundamnetal resonant mode. The parameters of the MSP is listed in Table 1. The numerical simulation is done for a mass load of M* = Δ*m/M* = 10^−5^. The solid line is the fitting curve.

To simulate the *S_m_*, let Δ*m/M* = 1 × 10^−5^ (*i.e.*, Δ*m* << *M*) at different locations (*x_c_*). The resonant frequency of the MSP with the mass load at different locations was determined using the numerical simulation with the properties and dimension listed in Table 1. Based on the resonant frequency, the *S_m_* of the MSP with the concentrated mass load at *x_c_* is obtained, which is plotted in Figure 1. From the data shown in Figure 1, one can find that the *S_m_* is symmetric about *x*_c_/*L* = 0.5 as expected. Additionally, the *S_m_* reaches the minimum value (*i.e.*, *S_m_* = 0) at *x*_c_/*L* = 0.5 and the maximum value (*S_m_*_,*max*_) at two ends (*i.e.*, *x*_c_/*L* = 0 and *x*_c_/*L* = 1) which are consistent with the expectations and the experimental results [8].

## 3. Results and Discussion

The location dependence of the mass sensitivity for the MSPs with a concentrated mass has been experimentally characterized [8]. The experimental results show that the change in the resonant frequency due to the attachment of a concentrated mass is dependent on both the location and the resonant mode [8]. Based on the results reported in [8], one can conclude that: (1) the *S_m_* for a concentrated mass at the node point (*i.e*., *φ*(*x*) = 0) is zero; (2) the *S_m_* reaches its maximum for a concentrated mass at the points at where the absolute value of the mode shape function, |φ(*x*)|, reaches its maximum; and (3) the *S_m_* for a concentrated mass at a location (*x*) monotonically increases with the |*φ*(*x*)|. That is, the *S_m_* has a tight relationship with the mode shape function. The physics behind this may be related to the influence of the mass load on the vibration behavior. When a mass load is attached to a resonator, there is a dragging force from the mass load to the resonator. The dragging force would be dependent on the acceleration of the mass load. For a resonator at one frequency with a concentrated mass at a location (*x_c_*), the bigger the |*φ*(*x_c_*)| is, the higher the acceleration is. The higher the acceleration is, the stronger the influence on the resonant behavior is. Therefore, one can expect that the *S_m_* for a concentrated mass at a location (*x_c_*) monotonically increases with the |*φ*(*x_c_*)|.

Based on these conclusions, an intuition take is that the *S_m_*(*x*) is proportional to |*φ*(*x*)|*^α^* (*α* > 0). If *α* is not an integer, the slope of the *S_m_* at the node point is not continuous. When α is an integer, the slope of the *S_m_* at the node point is not continuous if *α* (= 1, 3, 5, …) is odd number, but it is continuous if *α* (= 2, 4, 6, …) is an even number.

From the results shown in Figure 1, it is obtained that at two ends the *S_m_* reaches its maximum, *S_m,max_*, which is consistent with both the experimental results reported in [8] and the conclusion above since at two ends the |*φ*(*x*)| reaches the maximum. Here, *S_m,max_* = 93.96 Hz/ng, which is exactly the double of what $S_{m}^{uni}$ obtained using Equation (1). Interestingly, it is also found that the simulated *S_m_ versus x*_c_ curve shown in Figure 1 can be well fitted with:

(5) $$S_{m}\left(x_{c}\right)\;=\frac{S_{m,max}}{2}\left[1+\text{cos}\left(2\pi \frac{x_{c}}{L}\right)\right]=S_{m,max}cos^{2}\left(\pi \frac{x_{c}}{L}\right)$$

It is well known that the mode shape function, φ(*x*), for the fundamental resonant mode of an MSP without mass load can be written as [8]:

(6) $$\varphi \left(x\right)\;=cos\left(\pi \frac{x}{L}\right)$$

Based on the similarity between Equations (5) and (6), it is proposed that the *S_m_* of an AW device to a concentrated mass at *x_c_* can be determined by the corresponding mode shape function, *φ*(*x*), as:

(7) $$S_{m}\left(x_{c}\right)=C\varphi^{2}\left(x_{c})(\Delta m<<M\right)$$

where, *C* is a constant. The proposed Equation (7) is consistent with the intuition take from the experimental results reported in [8].

Considering a uniform mass load can be treated as a layer of concentrated mass, one should get:

(8) $$S_{m}^{uni}=\overset{L}{\underset{0}{\int }}S_{m}\left(x\right)dx$$

Combining Equations (7) and (8), the value of the constant “*C*” can be determined using the $S_{m}^{uni}$. Therefore, for an MSP with a concentrated mass at *x_c_*, the *S_m_* can be written as:

(9) $$S_{m}\left(x_{c}\right)\;=2S_{m}^{uni}cos^{2}\left(\pi \frac{x_{c}}{L}\right)$$

From Equation (9), one can find that $S_{m,max}=2S_{m}^{uni}$ for an MSP.

Based on above results, it is further introduced that Equations (7) and (8) can be used as a general methodology to determine the *S_m_* of any AW device with a concentrated mass. In other words, the *S_m_* of an AW device with a concentrated mass can be simply determined using the corresponding mode shape function, *φ*, for the AW device without mass load. To illustrate that this methodology can be used for all types of AW devices, two other types of AW devices (*i.e.*, cantilever and diaphragm) are studied here.

The cantilever has been widely studied as a high performance sensor platform in last two decades [6,9,10,21,22,23]. Various cantilevers, ranging in size from nanometers to micrometers, to even millimeters have been developed. For a one-end-fixed cantilever (length—*L*, width—*W*, thickness—H), the fundamental resonant frequency (*f*_0_) of the cantilever without mass load is:

(10) $$f_{0}=\frac{\lambda_{0}^{2}}{4\pi }\frac{H}{L^{2}}\sqrt{\frac{E}{3\rho \left(1-\sigma^{2}\right)}}(L>>W>>H)$$

where *λ*_0_ (= 1.87510) is the dimensionless eigenvalue [6]. The mode shape function of this resonant mode is [22]:

(11) $$\phi (x)=\cosh\frac{\lambda_{0}x}{L}-\cos\frac{\lambda_{0}x}{L}-\gamma_{0}\left(\sinh\frac{\lambda_{0}x}{L}-\sin\frac{\lambda_{0}x}{L}\right)$$

where $\gamma_{0}=\frac{\sinh\lambda_{0}-\sin\lambda_{0}}{\cosh\lambda_{0}+\cos\lambda_{0}}$, and the *x*-axis is defined as being along the length direction of the cantilever with *x* = 0 at the fixed end.

The $S_{m}^{uni}$ of a cantilever can be calculated using Equations (1) and (10). However, the *S_m_* of a cantilever with a concentrated mass is unknown. Due to the importance of the cantilever for the development of high-performance sensors, an effective-mass approach was used to determine the *S_m_* of a cantilever with a concentrated mass at the free end (*i.e.*, *x* = *L*) [24]. It should be mentioned that the *S_m_* for a cantilever with a concentrated mass at other locations cannot be determined even using this effective-mass approach. Based on this effective-mass approach with some approximations, it was concluded that the *S_m_* of a cantilever with a concentrated mass at the free end is [24]:

(12) Sm(L)≅f02*0.24267*M or Sm(L)Smuni≅4.12

**Figure 2:**
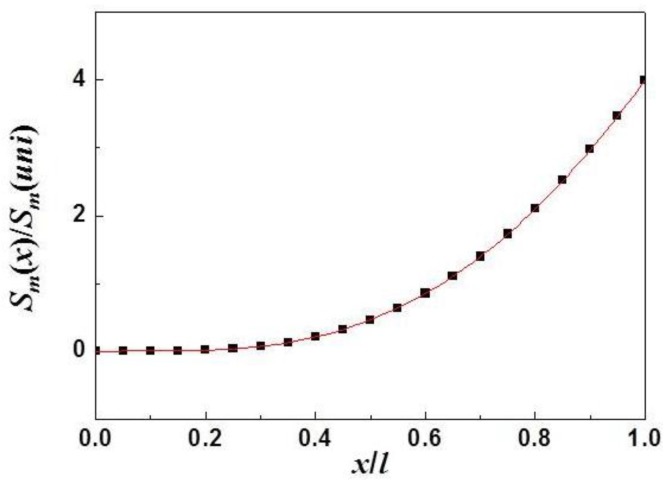
Mass sensitivity (*S_m_*) of a cantilever with a concentrated mass *versus* the location (*x*) of the mass, where *S_m_* is calculated using the methodology introduced here. The *y*-axis is normalized as $S_{m}\left(x\right)/S_{m}^{uni}$, where $S_{m}^{uni}$ is defined by Equations (1) and (10).

Using the methodology introduced here (*i.e.*, Equations (7) and (8)), the *S_m_* of a cantilever with a concentrated mass at *x_c_* can be easily written as:

(13) $$S_{m}\left(x_{c}\right)=\frac{S_{m}^{uni}}{\int_{0}^{L}\varphi^{2}\left(x\right)dx}\varphi^{2}\left(x_{c}\right)$$

where *φ*(*x*) is given by Equation (11) and $S_{m}^{uni}$ is determined by Equations (1) and (10). The location dependence of the *S_m_* for a cantilever with a concentrated mass described by Equation (13) is shown in Figure 2. Based on Equation (13), the highest *S_m_* is obtained for a cantilever with a concentrated mass load at the free end (*x* = *L*). That is, $S_{m,max}=S_{m}\left(L\right)$. This results in $\frac{S_{m}\left(L\right)}{S_{m}^{uni}}=4.0$, which is very close to the result (~4.12) obtained from the effective-mass approach described above. Considering the approximations used in the effective-mass approach, one may conclude that the methodology introduced here is correct and good enough to determine the *S_m_* of a cantilever with a concentrated mass at different locations.

Both MSPs and cantilevers studied above are one-dimensional devices. To further study the methodology introduced here, another AW device—two-dimensional diaphragm that has been used in the development of biological sensors [7,25]—is studied. A diaphragm can be either square or circular [7,26]. For a diaphragm with a square shape and a side length of “*a*” and a thickness of “*H*”, the fundamental resonant frequency (*f*_0_) is:

(14) $$f_{0}=\frac{\lambda_{0}^{2}}{4\pi }\frac{H}{a^{2}}\sqrt{\frac{E}{3\rho \left(1-\sigma^{2}\right)}}(a>>H)$$

where $\lambda_{0}^{2}=35.99$ [27]. Therefore, the $S_{m}^{uni}$ of a diaphragm can be calculated using Equations (1) and (14). The mode shape function of the fundamental resonant mode is [27]:

(15) $$\phi (x,y)=\sin\frac{\pi x}{a}*\sin\frac{\pi y}{a}$$

where *x* and *y* are along two directions: length (0 ≤ *x* ≤ *a*) and width (0 ≤ *y* ≤ *a*).

Using the methodology introduced here (*i.e.*, Equations (7) and (8)), the *S_m_* of a diaphragm with a concentrated mass at point (*x_c_*,*y_c_*) can be easily written as:

(16) $$S_{m}\left(x_{c},y_{c}\right)=\frac{S_{m}^{uni}}{\iint_{0}^{a}\varphi^{2}\left(x,y\right)dx\;dy}\varphi^{2}\left(x_{c},y_{c}\right)$$

Based on Equation (16), one can obtain that the *S_m_* reaches the maximum (*S_m,max_*) at the center: *x_c_* = *a*/2 and *y_c_* = *a*/2, which is consistent with experimental results. From Equation (16), it is also obtained that $\frac{S_{m,max}}{S_{m}^{uni}}=4.0$.

It should be mentioned that the *S_m_* of a diaphragm with a concentrated mass at the center was determined using the effective-mass approach. The results of the effective mass approach show that the *S_m_* of a diaphragm with a concentrated mass at the center is four times that for the diaphragm with a uniform mass load [25]. That is, for the concentrated mass at the center of a diaphragm, the conclusion of the effective-mass approach on the *S_m_* is the same as that of the new methodology introduced here. This again confirms the correctness of the new methodology introduced here.

**Application Remark:** The methodology introduced here is used to determine the *S_m_* for an ideally concentrated mass (*i.e.*, the volume is zero). In reality, a mass load is usually not an ideally concentrated mass, but is distributed over an area. If the area of a mass load is much smaller than the surface of the AW device, the methodology introduced here can be used as an approximation. If the area of a mass load is comparable with the surface of the AW device, the influence of the mass-load distribution has to be considered. In such case, one can treat a mass load over an area as an ideal mass load with a uniform distribution over the area. Therefore, the real mass sensitivity can be written as the integral of the *S_m_* over the area. For example, if a mass load fully covers a segment of an MSP or a cantilever from *x = x*_1_ to *x = x*_2_, the real mass sensitivity (*S_m,real_*) for this mass load should be:

(17) $$S_{m,real}=\frac{S_{m}^{uni}}{\int_{0}^{L}\varphi^{2}\left(x\right)dx}\int_{x_{1}}^{x_{2}}\varphi^{2}\left(x\right)dx$$

If the area of a mass load is smaller than the width of an MSP or a cantilever, the case cannot be treated as a one-dimensional case. However, an approximation approach can be used. For example, if a mass load covers an area of an MSP or a cantilever: along the length direction is from *x = x*_1_ to *x = x*_2_, along the width direction is wt% of the MSP/cantilever’s width, the real mass sensitivity (*S_m,real_*) for this mass load can be calculated approximately by:

(18) $$S_{m,real}=\left(wt\%\right)\frac{S_{m}^{uni}}{\int_{0}^{L}\varphi^{2}\left(x\right)dx}\int_{x1}^{x2}\varphi^{2}\left(x\right)dx$$

For a diagraph (*i.e.*, a two-dimensional case), the similar principle mentioned here can be used to calculate the sensitivity for a real mass load at different areas.

## 4. Conclusions

In conclusion, a numerical simulation approach was used to determine the influence of the mass-load location on the resonant frequency of the MSPs, which is used to determine the *S_m_* for the MSPs with a concentrated mass at different locations. Based on the results, it is introduced that the *S_m_* of an MSP with a concentrated mass can be simply determined using the mode shape function of the MSP without mass load. This methodology is further introduced as a general methodology to determine the *S_m_* of any AW device. That is, the *S_m_* of an AW device with a concentrated mass is proportional to the square of its mode shape function. This methodology was further proved using two widely used AW devices: (1) a one-dimensional cantilever; and (2) a two-dimensional diaphragm. Therefore, the *S_m_* for any AW device with a concentrated mass can be simply determined using the mode shape function of the AW device without mass load.
